# A Multicenter Survey of Type I Diabetes Mellitus in Chinese Children

**DOI:** 10.3389/fendo.2021.583114

**Published:** 2021-06-15

**Authors:** Ling Hou, Xiuzhen Li, Li Liu, Hanyang Wei, Feng Xiong, Hongwei Du, Yu Yang, Huifeng Zhang, Qin Zhang, Hui Yao, Junfen Fu, Xiaoli Yan, Lanwei Cui, Geli Liu, Tang Li, Shaoke Chen, Pin Li, Ying Xin, Xiangrong Liang, Baosheng Yu, Zhiya Dong, Ruimin Chen, Huamei Ma, Xinran Cheng, Feihong Luo, Chunxiu Gong, Wenhui Song, Xiaobo Chen, Zhixin Zhang, Xiangyun Peng, Guimei Li, Liyang Liang, Mireguli Maimaiti, Pik To Cheung, Xiaoping Luo

**Affiliations:** ^1^ Department of Pediatrics, Tongji Hospital, Tongji Medical College, Huazhong University of Science and Technology, Wuhan, China; ^2^ Department of Genetics and Endocrinology, Guangzhou Women and Children’s Medical Center, Guangzhou Medical University, Guangzhou, China; ^3^ Department of Endocrinology and Genetic, Henan Provincial Children’s Hospital, Zhengzhou, China; ^4^ Department of Endocrine and Genetic Metabolism Disease, Children’s Hospital of Chongqing Medical University, Chongqing, China; ^5^ Department of Pediatrics, The First Affiliated Hospital, Jilin University, Changchun, China; ^6^ Department of Endocrinology, Metabolism, and Genetics, Children’s Hospital of Nanchang University & Jiangxi Provincial Children’s Hospital, Nanchang, China; ^7^ Department of Pediatrics, The Second Affiliated Hospital of Hebei Medical University, Shijiazhuang, China; ^8^ Department of Endocrinology, Shenzhen Children’s Hospital, Shenzhen, China; ^9^ Department of Endocrinology, Wuhan Children’s Hospital, Wuhan, China; ^10^ Department of Endocrinology, Children’s Hospital, Zhejiang University School of Medicine, Hangzhou, China; ^11^ Department of Pediatrics Endocrinology, Xi’an Children’s Hospital, Xi’an, China; ^12^ Department of Pediatrics, The First Affiliated Hospital, Harbin Medical University, Harbin, China; ^13^ Department of Pediatrics, General Hospital of Tianjin Medical University, Tianjin, China; ^14^ Department of Pediatrics, Qingdao Women and Children’s Hospital, Qingdao University, Qingdao, China; ^15^ Department of Pediatrics, The Second Affiliated Hospital of Guangxi Medical University, Nanning, China; ^16^ Department of Endocrinology, Shanghai Children’s Hospital, Shanghai Jiao Tong University, Shanghai, China; ^17^ Department of Pediatrics Endocrinology, Shengjing Hospital of China Medical University, Shenyang, China; ^18^ Department of Endocrinology, Qilu Children’s Hospital, Shandong University, Jinan, China; ^19^ Department of Pediatrics, The Second Affiliated Hospital, Nanjing Medical University, Nanjing, China; ^20^ Department of Pediatrics, Ruijin Hospital, Shanghai Jiao Tong University, Shanghai, China; ^21^ Department of Endocrinology, Fuzhou Children’s Hospital, Fuzhou, China; ^22^ Department of Pediatrics, The First Affiliated Hospital, Sun Yat-sen University, Guangzhou, China; ^23^ Department of Endocrinology and Metabolism, Chengdu Women’s and Children’s Central Hospital, Chengdu, China; ^24^ Department of Pediatric Endocrinology and Inherited Metabolic Diseases, Children’s Hospital of Fudan University, Shanghai, China; ^25^ Endocrinology, Genetics, and Metabolism, Beijing Diabetes Center for Children and Adolescents, Medical Genetics Department, Beijing Children’s Hospital, Beijing, China; ^26^ Department of Pediatric Endocrinology, Shanxi Provincial Children’s Hospital, Taiyuan, China; ^27^ Department of Endocrinology, Children’s Hospital of Capital Institute of Pediatrics, Beijing, China; ^28^ Department of Pediatrics, China-Japan Friendship Hospital, Beijing, China; ^29^ Department of Endocrinology, Hunan Provincial Children’s Hospital, Changsha, China; ^30^ Department of Pediatrics, Shandong Provincial Hospital, Jinan, China; ^31^ Department of Pediatrics, Sun Yat-Sen Memorial Hospital, Sun Yat-Sen University, Guangzhou, China; ^32^ Department of Pediatrics, The First Affiliated Hospital, Xinjiang Medical University, Urumqi, China; ^33^ Paediatric Endocrinology, Genetics, and Metabolism, Virtus Medical Group and The University of Hong Kong, Hong Kong SAR, China

**Keywords:** type 1 diabetes, children, insulin, education, survey, diabetic ketoacidosis

## Abstract

**Purpose:**

To investigate the features and treatment status of children with type 1 diabetes mellitus (T1DM) in China.

**Methods:**

We recruited patients <14 years of age with T1DM from 33 medical centers in 25 major cities of China between January 2012 and March 2015. All patients completed a questionnaire that was conducted by their pediatric endocrinologists at all centers.

**Results:**

A total of 1,603 children (755 males and 848 females) with T1DM participated in this survey. Of these, 834 (52.03%) of the patients exhibited diabetic ketoacidosis (DKA) at onset, while 769 patients (47.97%) did not exhibit DKA (non-DKA) at onset. There was a higher proportion of females (55.71%) in the cohort of patients exhibiting DKA at onset than in the non-DKA cohort (49.33%). The mean age of patients exhibiting DKA at presentation was 7.12 ± 0.14 years; this was significantly younger than that in non-DKA group (7.79 ± 0.15 years; P < 0.005). The frequency of DKA in 3 years old, 3-7 years old, and 7 years old or more was 77.21%, 26.17%, and 37.62%, respectively. Upon initial diagnosis, 29.4%, 15.2% and 11.8% of patients showed positivity for glutamic acid decarboxylase antibody (GADA), Insulin autoantibodies (IAA), or islet cell antibody (ICA), respectively. During six months follow-up, 244 patients (15.21%) reported receiving insulin pump therapy, and more than 60% of patients monitored their blood glucose levels less than 35 times per week. Although the majority of patients had no problems with obtaining insulin, 4.74% of the children surveyed were not able to receive insulin due to financial reasons, a shortage of insulin preparations, or the failure of the parents or guardians to acquire the appropriate medicine.

**Conclusion:**

DKA is more common in very young children. Treatment and follow-up of T1DM in China still face very serious challenges.

## Introduction

Type 1 diabetes mellitus (T1DM) represents a common endocrine disease that can exert significant effects on children’s health. Data show that the incidence of T1DM is increasing around the world on an annual basis (1–3). However, there is very little information about the diagnosis and treatment of T1DM in Chinese children, even though there is a quarter of the world’s population in China. According to the atlas of diabetes published by the International Diabetes Federation (IDF), the incidence of T1DM in Asia is predominantly based on data arising from India (4). The incidence of T1DM remains unstable in China, with a mean increase of 14.2% annually in Shanghai between 1997 and 2011 (5). A T1DM registration survey, based in multiple centers, showed that the incidence of T1DM in children aged between 0 and 14 years was 1.9/100,000 (6). However, there is a significant shortage of information relating to the specific characteristics of T1DM in Chinese children, particularly from multiple centers.

This research investigated the features and treatment status of children with T1DM in China. The goal was to enhance understanding of this condition and therefore to facilitate the clinical management of T1DM in children.

## Materials and Methods

### Participants

This project was supported by the World Diabetes Foundation and Pediatric Endocrine & Genetic Metabolism Group of the Chinese Medical Association and was designed to investigate the clinical features of newly diagnosed T1DM children <14 years of age in 33 medical centers from 25 major cities, which cover of the 77% population in China. The survey was carried out between January 2012 and March 2015 and conducted by pediatric endocrinologists at all centers. The cities spanned from the southeast to the northwest of China, including economically developed, developing, and underdeveloped areas. The participating medical centers featured the best local medical institutions and were attended by top children’s endocrinologists. Consequently, the survey was able to achieve good coverage of the status of T1DM in children across China.

This study was approved by the Ethics Committee of Tongji Hospital, Tongji Medical College, Huazhong University of Science and Technology (Approval number: TJ-IRB201162) and adhered to the tenets of the Declaration of Helsinki. Written informed consent was obtained from the parents of all children included in this study.

### Questionnaire Survey

All newly diagnosed patients were hospitalized at their first visit. During hospitalization, all patients and parents completed the first questionnaire that was conducted by their pediatric endocrinologists. This questionnaire included birth history, infant feeding status, and the family history of T1DM, type 2 diabetes mellitus (T2DM), and gestational diabetes mellitus (GDM). Symptoms were recorded and the following examinations were performed: blood glucose; insulin; glycated hemoglobin (HbA1c); T1DM-related antibodies, including GADA, IAA, and ICA. We also performed thyroid function tests and antibodies, We did not record whether the patients required the pediatric intensive care unit (PICU) or not, or the duration of hospitalization.

In the next 6 months of follow-up, all the patients revisited their doctors (pediatric endocrinologists) at least one to two times at the clinic. All patients and their parents completed the second questionnaire conducted by their doctors for a range of further details including insulin preparations, mode of administration (MDI or pumps), times of blood glucose monitored per week, how the patients obtained insulin, sources of acquiring diabetes-related knowledge, and whether the patients received their drugs on time.

All children were diagnosed according to criteria for T1DM from American Diabetes Association (ADA). DKA was defined in the light of guideline published by the International Society of Pediatric and Adolescent Diabetes (ISPAD) (7).

### Statistical Analysis

Categorical data, including percentages and composition ratios, were tested by the Chi-squared test. Bonferroni adjustment were used for multiple comparisons. Measurement data, including blood glucose monitoring times and glycated hemoglobin levels, were compared by the *t* test for two groups. ANOVA were used for multiple comparisons. Differences were considered to be statistically significant if P<0.05.

## Research Results

### Patient Characteristics

A total of 1,603 children with T1DM including 755 males (47.1%) and 848 females (52.9%) were enrolled and all the patients and their parents finished the survey. None of the patients died during the study. The mean age at presentation was 7.6 ± 3.8 years. Most of the patients experienced onset at 3 to 12 years, of them, 23.22%, 24.70%, and 24.33%, were 3 to 6 years, 6 to 9 years, and 9 to 12 years, respectively. The proportions of children under 3 years of age and above 12 years of age were 13.72% and 13.91%.

With regard to feeding methods, the proportions of children receiving breastfeeding, artificial feeding, and mixed feeding, were 67.10%, 12.39%, and 20.51%, respectively. Children with a positive family history of diabetes, including T1DM or T2DM accounted for 21.54% of the total cohort. Analysis also showed that 2.87% of the children had a family history of GDM and 1.32% of the children had a family history of GDM and type 1 or type 2 diabetes.

### The Diagnosis and Treatment of Children With T1DM

#### The Symptoms at Presentation

At the initial diagnosis, 834 (52.03%) of patients presented with DKA and 769 (47.97%) did not present with DKA (non-DKA). Of the non-DKA patients, 649 (84.42%) patients presented with classical symptoms of polydipsia, polyuria, and weight loss. Only 32 cases (4.17%) were diagnosed during routine physical examinations and 88 (11.44%) cases were diagnosed when seeking medical attention for illness symptoms, such as fever, cough, vomiting, and diarrhea rather than diabetes symptoms.

All the patients received the T1DM-related antibodies tests, 625 patients (38.99%) were negative and 978 patients (61.01%) were positive. Upon initial diagnosis, the proportions of patients who were positive for GADA, IAA, and ICA, accounted for 29.4%, 15.2%, and 11.8%. The proportions of patients who were positive for one, two and all three of these antibodies, were 22.38%, 15.38%, and 1.20%, respectively. All patients had normal thyroid function and were negative for thyroid-related antibodies at presentation.

#### Comparative Analysis of Children With DKA and Non-DKA at Presentation

**Figure 1:**
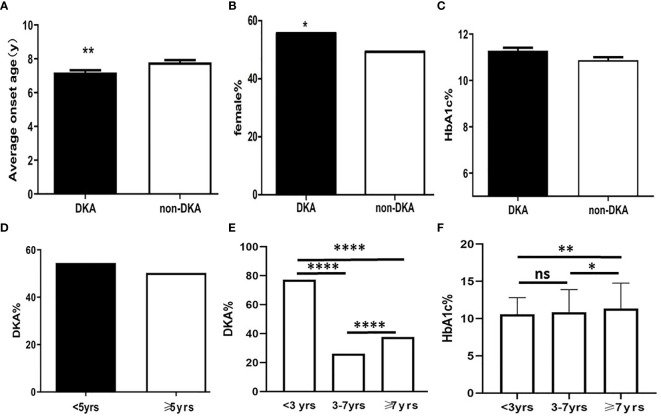
The diagram of predicators in T1DM children at presentation. **(A)** Average onset age between DKA and non-DKA groups; **(B)** Proportion of female between DKA and non-DKA groups; **(C)** HbA1c level between DKA and non-DKA groups; **(D)** Frequency of DKA between patients under 5 years old and over 5 years old; **(E)** DKA frequency of different age groups; **(F)** HbA1c level of different age groups *p < 0.05, **p < 0.01,****p < 0.0001. ns p > 0.05.

The mean age of patients exhibiting DKA at presentation was 7.12 ± 0.14 years; this was significantly younger than that in non-DKA group (7.79 ± 0.15 years; t = 2.97; P = 0.0031; **Figure 1A**). There was a significantly larger proportion of females in DKA group (55.71%) than that in non-DKA group (49.33%) (χ^2^ = 5.70; P = 0.017; **Figure 1B**).

At presentation, the mean HbA1c level was 11.09 ± 3.22% in the cohort; there was no significant difference between DKA group and non-DKA group with regards to HbA1c levels (11.21 ± 0.13% *vs*. 10.90 ± 0.13%; t= 1.72; P = 0.09; **Figure 1C**).

In this study, there was no significant difference in terms of the frequency of DKA when compared between the groups of children under 5 years of age and those over 5 years of age (χ^2^ = 2.2; P = 0.06; **Figure 1D**). The frequency of DKA in 3 years old, 3 to 7 years old, and 7 years old or more was 77.21%, 26.17%, and 37.62%, respectively. Analysis showed that there were significant differences between the three groups in terms of the frequencies of DKA (χ^2 =^ 127.88; P <0.0001; **Figure 1E**).

HbA1c levels at presentation in patients under 3 years of age, 3 to 7 years of age, and over 7 years of age, were 10.58 ± 2.28%, 10.85 ± 3.04%, and 11.35 ± 3.31%, respectively. There was significant difference among three groups (F=5.53, P=0.004). The HbA1c levels in group under 3 years of age was not significantly different compared to that in group 3 to 7 years of age (t=0.98; P = 0.33). However, there was a significant difference between children under 3 years of age and over 7 years of age (t=2.59; P=0.009) and 3 to 7 years of age and over 7 years of age (t=2.45; P=0.01) (**Figure 1F**).

#### Insulin Injection Therapy and Blood Glucose Monitoring

**Figure 2:**
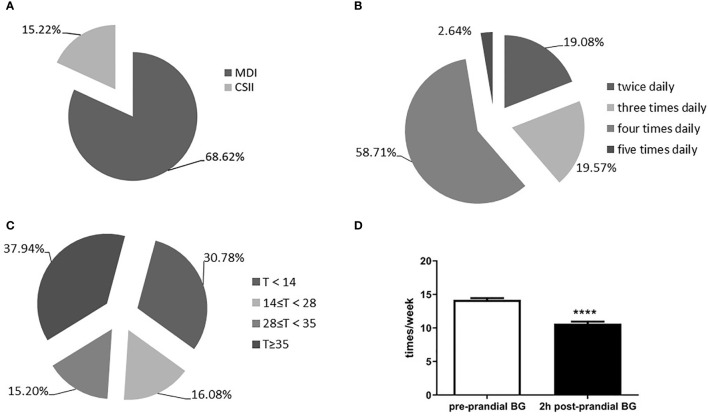
Insulin injection and blood glucose monitoring. **(A)** Intensive insulin injection scheme; **(B)** Proportion of different daily insulin subcutaneous injection programmes; **(C)** Times of self-monitoring of blood glucose per week; **(D)** Comparision of blood glucose monitoring times between pre-prandial blood glucose and 2h-post prandial blood glucose. MDI, multiple daily subcutaneous injections; CSII, continuous subcutaneous insulin infusion; T, times of self-monitoring of blood glucose ****p < 0.0001.

All children received insulin regimen including recombinant human insulin or insulin analogues decided by their doctors. After discharge, the patients selected drug regimen by their guardians and doctors. Total of 1,603 patients visited once, and 727 patients visited twice in the next 6 months follow-up. Data were collected till their last visit. Among them, 244 of 1,603 patients (15.21%) received a continuous and subcutaneous insulin infusion (CSII) with an insulin pump, while 1,100 (68.62%) were treated with a multiple daily injection (MDI) (**Figure 2A**). Among the group receiving daily injections, 798 cases (58.71%) received four injections per day; 266 cases (19.57%) and 259 cases (19.08%) received three and two injections per day, respectively (**Figure 2B**).

During follow-up, the survey showed that the proportions of patients monitoring blood glucose less than 14 times, 14 to 28 times, 28 to 35 times, and more than 35 times per week, were 30.78%, 16.08%, 15.20%, and 37.94%, respectively (**Figure 2C**). Pre-prandial blood glucose was checked significantly more frequently than 2-h post-prandial blood glucose (14.17 ± 0.28 times per week *vs.* 10.65 ± 0.28 times per week; t=8.8; *P* < 0.0001; **Figure 2D**).

### Insulin Available Access and Sources of Diabetes Education

All the patients received insulin therapy during their period of hospitalization. We were specifically interested in identifying the number of patients who were unable to acquire their insulin in time. Data analysis showed that 4.74% of the children surveyed could not receive insulin in time: 19.74% for financial reasons, 27.63% due to shortages of insulin preparations, 26.32% due to the failure of the parents or guardians to obtain the medicine in time, and 26.32% for other reasons.

**Figure 3:**
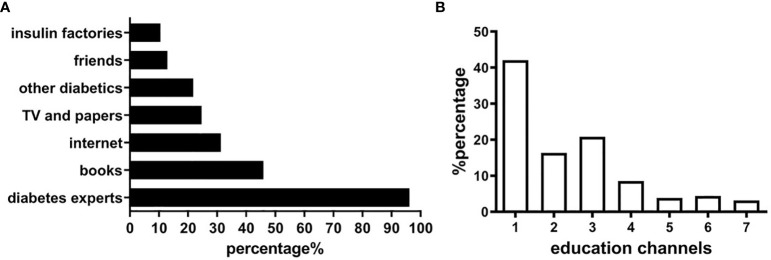
Diabetes education in patients and guardians. **(A)** Relevant sources for diabetes education. **(B)** Education channels of obtaining diabetes knowledge.

The patients and their guardians were able to receive diabetes education from their doctor when they visited at clinic. In addition, most of parents and guardians were able to receive education from other sources. Among all the patients and guardians surveyed, 96.16% received diabetes education from health workers (including pediatric endocrinologists and diabetes nurses), 45.89% from books, 31.25% from the internet, 24.64% from newspapers and televisions, 21.79% from other patients with diabetes, and 10.54% from the manufacturers of insulin (**Figure 3A**). Further analysis showed that 42.05% of patients obtained their knowledge *via* one channel, 16.34% *via* 2 channels, and 40.71% *via* 3 or more channels (**Figure 3B**).

## Discussion

According to data released by IDF in 2017, 1.1 million children and adolescents (<20 years of age) have T1DM around the world and 80% of these children reside in low- and middle-income countries (8). In recent years, the number of children with T1DM has been rising steadily (5, 9). However, prior to the present study, little was known about the status of Chinese children with T1DM.

In this cohort, children under 3 years of age accounted for 13.72%, which was higher than 3.61% reported from the United States in 2009 (10). The proportion of children older than 9 years in China was very similar to that reported in the United States in 2009 (10). Children with a positive family history of diabetes, including T1DM and T2DM, accounted for 21.54% of the total cohort. This suggested that genetic factors might represent a high-risk factor for the development of T1DM in Chinese children. A previous study, undertaken in Asturias, Spain showed that 14.4% of children with T1DM under 14 years of age had a family history of T1DM, and 29.4% of children had a family history of T2DM (11). The differences between these two countries need to be investigated further. It is possible that race and environmental factors might underlie these observations.

At the first diagnosis, 978 cases (61.01%) tested positive for T1DM-related antibodies. The proportions of children who were positive for GADA, IAA, and ICA, were 29.4%, 15.2%, and 11.8%, respectively. In a previous Brazilian study, the frequency of antibody positivity was 56.8% for GAD, 43.7% for ICA, and 26.3% for IAA (12). Of the 241 T1DM patients presenting at <15 years of age in Melbourne, Australia, 58.9% of cases were GAD seropositive and 42.3% were IAA seropositive. Factors associated with elevated IAA antibodies included younger age and the red hair phenotype. Factors associated with elevated GAD antibodies included a lower birthweight and recent eczema (13). This study showed antibodies positive were lower in Chinese children with T1DM than that in other countries. In Weng’s study (6), 73.5% of the T1DM patients’ age are 0 to 14 years had at least one positive test result for diabetes autoantibodies, including GAD, the insulin antigen 2, ICA, IAA, and Zn8. But there was no specific percentage of each antibody positive presented in this report. In our study, the reasons for the low positive rate of diabetes-related antibodies may be: 1) Laboratory standardization of each center was not uniform which may lead to false negatives. 2) The patients were newly diagnosed, and the course of the disease was not long. 3) The age distribution were different and antibodies may be related to age. In general, the difference in the frequency of antibodies positivity between Chinese and other populations need further study.

DKA is a serious complication of T1DM and an important cause of T1DM-related death in children (14–16). The incidence of DKA is highest in low- and middle-income countries (17), but remains unacceptably high even in high resource countries around the world. In this study, more than half of the children (52.03%) surveyed initially presented with DKA; this was higher than that reported previously in French children (43.9%), Polish children (23%) and Kuwaiti children (35.6%) (18–20). Studies arising from the USA also reported that 34-46% of patients presented with DKA (21, 22). The incidence of DKA was reported to be much higher in Chinese children with T1DM (6) than children in other developed countries and was similar to that of India (>50%) (23). A single-center study in Pakistan reported that 83% of children with T1DM were admitted to the ICU as a direct result of DKA (16). Therefore, in countries with a low incidence of T1DM (such as China), the awareness for DKA tends to be low while the rate of DKA is high. In contrast, the rate of DKA is much lower in countries with a high incidence of T1DM. This may likely reflect education and public awareness. ISPAD/the Life for a Child Program (LFAC) emphasis on increasing awareness not only of general population with posters in schools and markets has been successful in LFAC sites around the world in many low and medium resource countries but also in high resource countries like Italy and Canada for same reasons. Focused availability of ongoing pediatric and adolescent DKA lectures to emergency room and pediatricians and general practitioners helps in addition to the poster campaigns (24).

According to a previous study, DKA was more frequently encountered in children <5 years of age (25). Another study reported that severe DKA was more common in children <3 years of age (26). At presentation, the mean age in DKA group was significantly lower than that in non-DKA group. This suggested that younger children are more likely to present with DKA as the initial symptom and should be assessed carefully in clinical practice (14). This study also indicated that the frequency of DKA was significantly higher in females than in males. A previous study reported that the incidence of DKA was significantly higher in females than in males after treatment (27). Nonetheless, the specific reasons for the higher frequency of DKA in girls than boys have to be elucidated. Iovane et al. reported that children under 5 years old had higher frequency of DKA (25). In the present study, we did not identify differences in the frequency of DKA between children <5 years of age and those over 5 years of age. We further analyzed the frequency of DKA in groups under 3 years of age, 3 to 7 years of age, and 7 years of age or older. The data indicated that under 3 years of age were more likely to present with DKA than older children and this was in accordance with Bui’s report (28). After 3 years of age, the frequency of DKA has been shown to be independent from age (28). Szypowska reported that Children <3 and <5 years of age were at higher risk of DKA but not of severe DKA (29). Several potential reasons would explain why younger children can be at a higher risk of DKA. Early symptoms of diabetes may be missed in young children because frequent vomiting and increased work of breathing which are frequently observed symptoms may be expected by health care worker as well as parents to be from other explanations. Younger children are less likely to have free access to fluids and may be more severely dehydrated and acidotic at presentation (26).

There was no significant difference between the DKA group and the non-DKA group in terms of HbA1c level. Further analysis showed that younger children actually had lower HbA1c levels than older children. In younger children with T1DM the medial care providers need to be more vigilant about the possibility of DKA and its complications like cerebral edema and death even if the HbA1c level was not so high. The underlying related mechanism between DKA and HbA1c in different age was uncertain but may reflect actual duration of symptoms pre-diagnosis.

Intensive insulin therapy is very important for children with T1DM (30). The Diabetes Control and Complications Trial has demonstrated that intensive therapy effectively delays the onset and slows the progression of diabetic retinopathy and nephropathy when initiated in adolescent subjects (31, 32). In this study, the patients were still predominantly receiving multiple daily subcutaneous injections of insulin. The proportion of patients using insulin pumps was only 15.21%, much lower than that reported for developed countries: 50% in patients < 6 years of age in the United States, and 74% in Germany and Australia (33). Most studies suggest that insulin pump therapy is more conducive to blood glucose control and can achieve lower HbA1c levels, less hypoglycemia (especially nocturnal hypoglycemia) and greater time in range so minimizing long-term complications (34–36). The use of insulin pumps might be related to the level of economic development in China or lack of universal health insurance coverage. Most of the users we surveyed were from developed regions so deductible amounts not just for insulin and blood glucose strips but also for high-cost technology like pumps and sensors may be key factors.

The timely treatment of childhood diabetes and the availability of medicines depend largely on parents or guardians. During the survey period, inpatient diabetic patients in China were covered by medical insurance for major illnesses, while outpatients were not covered by medical insurance. Therefore, the patients had to pay for insulin preparations, blood glucose testing strips, meters, pumps. In this study, approximately 4.74% of children could not receive their insulin within an acceptable period of time, although most children could receive insulin treatment in time. Among the group children, the key factors were due to financial insufficiency, shortages of insulin preparations, and failure of the parents or guardians to obtain the medicine in time. These data suggested that the government and medical insurance companies should pay more attention to enhancing patient follow-up and insulin supply.

Blood glucose monitoring plays an important role to lower the rate of T1DM-related complications during follow-up. In the present study, approximately 37.94% of Chinese children were able to complete standard blood glucose monitoring (more than 35 times per week) (37). However, up to 30.78% failed to check their blood glucose timely or only check occasionally (<14 times per week). Insufficient blood glucose monitoring might result in less home adjustments and high occurrence of hypoglycemia. These data indicated that health education still need to be enhanced. Most children and guardians checked their pre-prandial blood glucose but tended to ignore the 2-h post-prandial blood glucose. One key reason was that most of children were attending kindergartens and schools during the day. Another reason was that the parent and guardians were likely also often fearful of societal or peer negative responses so kept the diagnosis secret from teachers, coaches and others at some risk to the child with diabetes. In addition, insulin type such as older or analogue affect the glycemic control and less severe or lower time below range overall occurs. More details should include the information (38, 39).

In China, diabetes education mainly relies on the medical staff, specifically who are engaged in the care of diabetes. This survey showed that the vast majority of children and guardians still received diabetes-related information through medical workers such as their doctors and nurses but no dieticians or social workers/psychologists with a small number obtaining information *via* multiple sources. In recent years, the increasing application of communication networks has led to the increased application of distance education in the management of chronic diseases. Diabetes camps, group diabetes outpatient teaching of self-management of blood glucose monitoring and diabetes-related health education *via* internet-based sources has improved dramatically over recent years (40, 41). Although the internet for diabetes education is increasing, its supervision and standardization need to be strengthened and resourced with Chinese language and society/food patterns, school staff awareness and participation and better available and resourced multidisciplinary health care team (42).

This study has certain limitations that need to be considered. Firstly, since the participating hospitals or centers were located in regions of various economic levels, the medical resources located in these regions may well exhibit variation in terms of quality and quantity. It is possible that these factors may result in bias. Secondly, this study did not survey all patients from the 25 cities. Consequently, we were unable to calculate the precise incidence of T1DM in China. Thirdly, since HbA1c levels were not obtained regularly, we were unable to demonstrate how the levels of HbA1c change. Cost barriers and other factors which explain the lack of outpatient diabetes follow-up care coupled with costs of paying for more frequent lab work such as HbA1c will likely need to be addressed as well after therapy.

In summary, this multi-center study provided an enhanced understanding of the characteristics of children with T1DM in China and indicated that the control and management of T1DM in children remains a formidable challenge.

## Data Availability Statement

The data analyzed in this study is subject to the following licenses/restrictions: for article data analysis only. Requests to access these data sets should be directed to houlingtj@163.com.

## Ethics Statement

Upon detailed explanation of the study protocol, all participants signed the informed consent.

## Author Contributions

XiaL designed and organized the study and cowrote the first draft of the manuscript. LH assisted organization of the study and wrote the draft of the manuscript. All authors organized the study in their study regions and contributed to the data collection. All authors listed have made a substantial, direct, and intellectual contribution to the work and approved it for publication.

## Funding

This research was supported by grants from the World Diabetes Foundation (reference: WDF11-617) and the Ministry of Science and Technology (reference: 2017ZX09304022).

## Conflict of Interest

The authors declare that the research was conducted in the absence of any commercial or financial relationships that could be construed as a potential conflict of interest.

## References

[1] Whiting DR Guariguata L Weil C Shaw J. IDF Diabetes Atlas: Global Estimates of the Prevalence of Diabetes for 2011 and 2030. Diabetes Res Clin Pract (2011) 94:311–21. 10.1016/j.diabres.2011.10.029 22079683

[2] Guariguata L Whiting DR Hambleton I Beagley J Linnenkamp U Shaw JE. Global Estimates of Diabetes Prevalence for 2013 and Projections for 2035. Diabetes Res Clin Pract (2014) 103:137–49. 10.1016/j.diabres.2013.11.002 24630390

[3] Tuomilehto J. The Emerging Global Epidemic of Type 1 Diabetes. Curr Diabetes Rep (2013) 13:795–804. 10.1007/s11892-013-0433-5 24072479

[4] IDF Diabetes Atlas Group. Update of Mortality Attributable to Diabetes for the IDF Diabetes Atlas: Estimates for the Year 2013. Diabetes Res Clin Pract (2015) 109:461–5. 10.1016/j.diabres.2015.05.037 26119773

[5] Zhao Z Sun C Wang C Li P Wang W Ye J. Rapidly Rising Incidence of Childhood Type 1 Diabetes in Chinese Population: Epidemiology in Shanghai During 1997-2011. Acta Diabetol (2014) 51:947–53. 10.1007/s00592-014-0590-2 24777734

[6] Weng J Zhou Z Guo L Zhu D Ji L Luo X. T1D China Study Group. Incidence of Type 1 Diabetes in China, 2010-13: Population Based Study. BMJ (2018) 360:j5295. 10.1136/bmj.j5295 29298776 PMC5750780

[7] Wolfsdorf JI Allgrove J Craig ME Edge J Glaser N Jain V. ISPAD Clinical Practice Consensus Guidelines 2014. Diabetic Ketoacidosis and Hyperglycemic Hyperosmolar State. Pediatr Diabetes (2014) 15:154–79. 10.1111/pedi.12165 25041509

[8] International Diabetes Federation. International Diabetes Federation Diabetes Atlas, 9th ed. (2017).

[9] Fu JF Liang L Gong CX Xiong F Luo FH Liu GL. Status and Trends of Diabetes in Chinese Children: Analysis of Data From 14 Medical Centers. World J Pediatr (2013) 9:127–34. 10.1007/s12519-013-0414-4 23677831

[10] Dabelea D Mayer-Davis EJ Saydah S Imperatore G Linder B Divers J. SEARCH for Diabetes in Youth Study. Prevalence of Type 1 and Type 2 Diabetes Among Children and Adolescents From 2001 to 2009. JAMA (2014) 311:1778–86. 10.1001/jama.2014.3201

[11] Álvarez SO Rodríguez MDRR Sánchez RL Pérez FA Rodríguez EO. Type 1 Diabetes Mellitus Prevalence and Care in Children Under 15 Years Old in Asturias. Endocrinol Diabetes Nutr (2019) 6:188–94. 10.1016/j.endinu.2018.08.008 30413391

[12] de Souza LCVF Kraemer GdeC Koliski A. Diabetic Ketoacidosis as the Initial Presentation of Type 1 Diabetes in Children and Adolescents: Epidemiological Study in Southern Brazil. Rev Paul Pediatr (2019) 38:e2018204. 10.1590/1984-0462/2020/38/2018204 31778415 PMC6909258

[13] Ponsonby AL Pezic A Cameron FJ Rodda C Ellis JA Kemp AS. Phenotypic and Environmental Factors Associated With Elevated Autoantibodies at Clinical Onset of Paediatric Type 1 Diabetes Mellitus. Results Immunol (2012) 2:125–31. 10.1016/j.rinim.2012.06.002 PMC3862385 24371576

[14] Rewers A Klingensmith G Davis C Petitti DB Pihoker C Rodriguez B. Presence of Diabetic Ketoacidosis at Diagnosis of Diabetes Mellitus in Youth: The Search for Diabetes in Youth Study. Pediatrics (2008) 121:e1258–66. 10.1542/peds.2007-1105 18450868

[15] Usher-Smith JA Thompson M Ercole A Walter FM. Variation Between Countries in the Frequency of Diabetic Ketoacidosis at First Presentation of Type 1 Diabetes in Children: A Systematic Review. Diabetologia (2012) 55:2878–94. 10.1007/s00125-012-2690-2 PMC3464389 22933123

[16] Jawaid A Sohaila A Mohammad N Rabbani U. Frequency, Clinical Characteristics, Biochemical Findings and Outcomes of DKA at the Onset of Type-1 DM in Young Children and Adolescents Living in a Developing Country - An Experience From a Pediatric Emergency Department. J Pediatr Endocrinol Metab (2019) 32:115–9. 10.1515/jpem-2018-0324 30699071

[17] Hwang CK Han PV Zabetian A Ali MK Narayan KM. Rural Diabetes Prevalence Quintuples Over Twenty-Five Years in Low- and Middle-Income Countries: A Systematic Review and Meta-Analysis. Diabetes Res Clin Pract (2012) 96:271–85. 10.1016/j.diabres.2011.12.001 22261096

[18] Choleau C Maitre J Filipovic Pierucci A Elie C Barat P Bertrand AM. AJD Study Group. Ketoacidosis at Diagnosis of Type 1 Diabetes in French Children and Adolescents. Diabetes Metab (2014) 40:137–42. 10.1016/j.diabet.2013.11.001 24332018

[19] Chumięcki M Prokopowicz Z Deja R Jarosz-Chobot P. Frequency and Clinical Manifestation of Diabetic Ketoacidosis in Children With Newly Diagnosed Type 1 Diabetes. Pediatr Endocrinol Diabetes Metab (2013) 19:143–7. 25612814

[20] Shaltout AA Channanath AM Thanaraj TA Omar D Abdulrasoul M Zanaty N. Ketoacidosis at First Presentation of Type 1 Diabetes Mellitus Among Children: A Study From Kuwait. Sci Rep (2016) 22:27519. 10.1038/srep27519 PMC4916451 27328757

[21] Klingensmith GJ Tamborlane WV Wood J Haller MJ Silverstein J Cengiz E. Pediatric Diabetes Consortium. Diabetic Ketoacidosis at Diabetes Onset: Still an All Too Common Threat in Youth. J Pediatr (2013) 162:330–4. 10.1016/j.jpeds.2012.06.058 22901739

[22] Rewers A Dong F Slover RH Klingensmith GJ Rewers M. Incidence of Diabetic Ketoacidosis at Diagnosis of Type 1 Diabetes in Colorado Youth, 1998-2012. JAMA (2015) 313:1570–2. 10.1001/jama.2015.1414 25898057

[23] Kanwal SK Bando A Kumar V. Clinical Profile of Diabetic Ketoacidosis in Indian Children. Indian J Pediatr (2012) 79:901–4. 10.1007/s12098-011-0634-3 22207489

[24] Holder M Ehehalt S. Significant Reduction of Ketoacidosis at Diabetes Onset in Children and Adolescents With Type 1 Diabetes—the Stuttgart Diabetes Awareness Campaign, Germany. Pediatr Diabetes (2020) 21:1227–31. 10.1111/pedi.13064 32579294

[25] Iovane B Cangelosi AM Bonaccini I Di Mauro D Scarabello C Panigari A. Diabetic Ketoacidosis at the Onset of Type 1 Diabetes in Young Children Is it Time to Launch a Tailored Campaign for DKA Prevention in Children <5 Years? Acta BioMed (2018) 89:67–71. 10.23750/abm.V89i1.6936 29633745 PMC6357617

[26] Glackin S Metzger D Hanas R Chanoine J-P. Is Age a Risk Factor for Cerebral Edema in Children With Diabetic Ketoacidosis? A Literature Review. Can J Diabetes (2020) 44:111–8. 10.1016/j.jcjd.2019.04.013 31311730

[27] Maahs DM Hermann JM Holman N Foster NC Kapellen TM Allgrove J. National Paediatric Diabetes Audit and the Royal College of Paediatrics and Child Health, the DPV Initiative, and the T1D Exchange Clinic Network. Rates of Diabetic Ketoacidosis: International Comparison With 49,859 Pediatric Patients With Type 1 Diabetes From England, Wales, the U.S., Austria, and Germany. Diabetes Care (2015) 38:1876–82. 10.1007/s12098-011-0634-3 26283737

[28] Bui H To T Stein R Fung K Daneman D. Is Diabetic Ketoacidosis at Disease Onset a Result of Missed Diagnosis? J Pediatr (2010) 156(3):472–7. 10.1007/s12098-011-0634-3 19962155

[29] Szypowska A Ramotowska A Grzechnik-Gryziak M Szypowski W Pasierb A Piechowiak K. High Frequency of Diabetic Ketoacidosis in Children With Newly Diagnosed Type 1 Diabetes. J Diabetes Res (2016) 2016:9582793. 10.1155/2016/9582793 26783540 PMC4691462

[30] Brink SJ Lee WRW Pillay K Kleinebreil L. Diabetes in Children and Adolescents. Baegsvard, Denmark: NovoNordisk A/S (2010).

[31] Effect of Intensive Diabetes Treatment on the Development and Progression of Long-Term Complications in Adolescents With Insulin-Dependent Diabetes Mellitus: Diabetes Control and Complications Trial. Diabetes Control and Complications Trial Research Group. J Pediatr (1994) 125:177–88. 10.1016/S0022-3476(94)70190-3 8040759

[32] Danne T Phillip M Buckingham BA Jarosz-Chobot P Saboo B Urakami T. ISPAD Clinical Practice Consensus Guidelines 2018: Insulin Treatment in Children and Adolescents With Diabetes. Pediatr Diabetes (2018) 19(Suppl 27):115–35. 10.1111/pedi.12718 29999222

[33] Maahs DM Hermann JM DuBose SN Miller KM Heidtmann B DiMeglio LA. DPV Initiative; T1D Exchange Clinic Network. Contrasting the Clinical Care and Outcomes of 2,622 Children With Type 1 Diabetes Less Than 6 Years of Age in the United States T1D Exchange and German/Austrian DPV Registries. Diabetologia (2014) 57:1578–85. 10.1007/s00125-014-3272-2 24893863

[34] Karges B Schwandt A Heidtmann B Kordonouri O Binder E Schierloh U. Association of Insulin Pump Therapy vs Insulin Injection Therapy With Severe Hypo, Ketoacidosis, and Glycemic Control Among Children, Adolescents, and Young Adults With T1DM. JAMA (2017) 318:1358–66. 10.1001/jama.2017.13994 PMC5818842 29049584

[35] Maiorino MI Casciano O Della Volpe E Bellastella G Giugliano D Esposito K. Reducing Glucose Variability With Continuous Subcutaneous Insulin Infusion Increases Endothelial Progenitor Cells in Type 1 Diabetes: An Observational Study. Endocrine (2016) 52:244–52. 10.1007/s12020-015-0686-7 26184417

[36] Alemzadeh R Palma-Sisto P Holzum M Parton E Kicher J. Continuous Subcutaneous Insulin Infusion Attenuated Glycemic Instability in Preschool Children With Type 1 Diabetes Mellitus. Diabetes Technol Ther (2007) 9:339–47. 10.1089/dia.2006.0038 17705689

[37] Chinese Diabetes Society. Guidelines for the Clinical Application of Glucose Monitoring in China (2015 Edition). Chin J Diabetes Mellitus (2015) 7(10):603–13. 10.3760/cma.j.issn.1674-5809.2015.10.004

[38] Brink SJ. Diagnosis and Management of Type 1 Diabetes Mellitus in Children Adolescents and Young Adults. In: Velea I Paul C Brink SJ, editors. Pediatric Endocrinology and Diabetes 2018 Update. Timisoara, Romania: Editura Mirton (2018). p. 11–98.

[39] Brink SJ. 2015 Past, Present and Future Insulin Update. In: Velea I Paul C Brink SJ, editors. Update in Pediatric Endocrinology and Diabetes. Timisoara, Romania: Mirton (2015). p. 61–96.

[40] Mulvaney SA Rothman RL Wallston KA Lybarger C Dietrich MS. An Internet-Based Program to Improve Self-Management in Adolescents With Type 1 Diabetes. Diabetes Care (2010) 33:602–4. 10.2337/dc09-1881 PMC2827516 20032275

[41] Raiff BR Dallery J. Internet-Based Contingency Management to Improve Adherence With Blood Glucose Testing Recommendations for Teens With Type 1 Diabetes. J Appl Behav Anal (2010) 43:487–91. 10.1901/jaba.2010.43-487 PMC2938951 21358907

[42] Brink SJ. Pediatric and Adolescent Multidisciplinary Diabetes Team Care. Pediatr Diabetes (2010) 11:289–91. 10.1111/j.1399-5448.2010.00702.x 20646238

